# Applying Cognitive Learning Strategies to Enhance Learning and Retention in Clinical Teaching Settings

**DOI:** 10.15766/mep_2374-8265.10850

**Published:** 2019-11-01

**Authors:** Ariel S. Winn, Lisa DelSignore, Carolyn Marcus, Laura Chiel, Eli Freiman, Diane Stafford, Lori Newman

**Affiliations:** 1 Assistant Professor of Pediatrics, Harvard Medical School; 2 Associate Program Director for the Boston Combined Residency Program, Division of General Pediatrics, Department of Pediatrics, Boston Children's Hospital; 3 Assistant Professor in Pediatrics, Tufts University School of Medicine; 4 Assistant Professor, Department of Pediatrics, Floating Hospital for Children at Tufts Medical Center; 5 Instructor in Pediatrics, Harvard Medical School; 6 Chief Resident, Boston Combined Residency Program, Boston Medical Center; 7 Chief Resident, Boston Combined Residency Program, Boston Children's Hospital; 8 Clinical Professor of Pediatrics, Stanford University School of Medicine; 9 Associate Program Director, Pediatric Endocrinology Fellowship, Lucile Packard Children's Hospital Stanford; 10 Director of Professional Development, Department of Medical Education, Boston Children's Hospital

**Keywords:** Cognitive Learning Strategies, Active Learning, Memory Science

## Abstract

**Introduction:**

Cognitive learning strategies are strategies that improve a learner's ability to process information more deeply, transfer and apply information to new situations, and result in enhanced and better-retained learning.

**Methods:**

We developed an interactive workshop for a national conference of pediatric educators to teach five cognitive learning strategies. The specific strategies were (1) spaced retrieval practice, (2) interleaving, (3) elaboration, (4) generation, and (5) reflection. Each strategy was taught using an active learning exercise. We evaluated the effectiveness of the workshop through a commitment-to-change exercise in which we asked participants to commit to making a change in their teaching as it related to the workshop and then queried them 6 weeks later about their implementation successes and barriers.

**Results:**

Of the 161 participants registered for the workshop, 52 completed the voluntary workshop evaluation. All 52 participants committed to making a change in their teaching as a result of the workshop. Of those 52 participants, 24 completed the 6-week follow-up survey. Eighty-two percent of those respondents (*n* = 18) reported implementing a change based on the workshop, with 77% of respondents implementing a change that they had committed to directly after the workshop and 55% implementing a change that they had not originally committed to at the end of the workshop.

**Discussion:**

This workshop successfully led to behavioral change in the teaching of cognitive learning strategies. We anticipate that this will lead to improved learning among the trainees whom participants teach.

## Educational Objectives

By the end of this interactive workshop, learners will be able to:

1. Identify and describe five cognitive learning strategies.
2. Identify clinical teaching opportunities to apply cognitive learning strategies.
3. Implement different cognitive learning strategies in various clinical teaching settings.

## Introduction

Medical researchers project that the collective body of medical information will double every 73 days by 2020.^1^ It is incumbent on medical schools and clinical training programs to help students and trainees learn to absorb, organize, store, and retrieve this vast amount of information. Historically, students and trainees have been taught using passive learning strategies, such as rereading, highlighting, and cramming, along with attending lectures, conferences, or grand rounds as inactive participants. These passive learning strategies can be effective for short-term information recall, leading to the illusion of knowledge mastery, but are rarely effective in producing sustained learning.^2–7^

In contrast, there is emerging literature, popularized by the book *Make It Stick: The Science of Successful Learning*,^4^ indicating that applying cognitive learning strategies in active learning environments can help achieve more productive and sustained learning.^4–9^ Cognitive learning strategies are strategies that improve a learner's ability to process information more deeply, transfer and apply information to new situations, and result in enhanced and better-retained learning.^6,10–11^ These learning strategies engage learners in activities in which they are responsible for performing tasks while thinking about what they are learning and why they have reached particular solutions.^4–9,12–14^ There is solid evidence that routine integration of these strategies coupled with daily active learning practice results in higher-order and sustained learning outcomes.^15–18^

Although some medical educators have started to adopt active learning practices in the classroom setting, including the integration of problem-based learning, team-based learning, and flipped classroom models,^12,19–22^ many faculty have not received instruction on cognitive learning strategies.^12^ As more students enter medical school with exposure to cognitive learning strategies from their undergraduate college experiences, they will expect to engage in active learning using these strategies. Moreover, learners have increasingly begun to accept that delayed gratification and desirable difficulty lead to sustained retention of newly acquired knowledge and skills.^4,23–25^ Health professional students should therefore expect their faculty to be well trained in cognitive learning strategies.

In this workshop, participants learn the theory behind five specific evidence-based cognitive learning strategies. We identified these five strategies, after an extensive search of the literature, as valuable, practical, and easy to adopt within one's own teaching practice while greatly improving learning and retention of information. The five cognitive learning strategies addressed in this workshop include spaced retrieval practice, interleaving, elaboration, generation, and reflection^2,4,6,8,9,12^ (see Appendix A for definitions). Participants apply these cognitive learning strategies using concrete examples (both clinical and nonclinical) and engage in active group discussion and authentic practice. To encourage behavior change, participants then determine how to integrate these cognitive learning strategies into their current clinical teaching settings. This workshop provides a platform for health care professional educators to gain an understanding of five evidence-based cognitive learning strategies, apply these strategies, and then determine ways to incorporate cognitive learning into their teaching to promote knowledge gain, retention, and learner satisfaction. This workshop contributes to and enhances the existing literature on cognitive learning strategies, as it allows participants to practice each strategy and then determine practical ways they can incorporate the strategies when teaching in their own medical field or clinical discipline. Other workshops have focused primarily on questions, small-group discussions, or role-play as a way of teaching other active learning strategies based in cognitive learning theory.^26–30^ We know of no other source that provides this comprehensive educational experience.

## Methods

### Session Description and Implementation

We planned and presented this 90-minute workshop for the 2018 annual national meeting of the Association of Pediatric Program Directors (APPD). This workshop was adapted from a similar half-day retreat that we originally constructed for the Boston Children's Hospital Academy for Teaching and Educational Innovation and Scholarship, an interprofessional academy inclusive of self-identified clinician-educators (trainees, faculty, and health care professional staff) from all pediatric departments at Boston Children's Hospital.

A total of 161 participants registered for the APPD workshop. Registration information collected by the APPD organization showed that participants included pediatric residents, chief residents, and fellows, as well as junior and senior attending faculty members. No specific prerequisite knowledge of active learning or cognitive learning strategies was required. Our workshop was facilitated according to the outline and time line in the Table.

**Table.** Workshop Outline and Time Line

Time	Topic/Activity	Facilitator Responsible	Materials
5 minutes	Brief introduction to workshop objectives and facilitators	All facilitators	Microphones
15 minutes	Interactive didactic session on cognitive learning strategies (Appendix B)	One facilitator	Laptop and projector for the PowerPoint presentation, microphones
50 minutes (10-minute sessions)	Small-group sessions: • Spaced retrieval practice (Appendix C) • Interleaving (Appendix D) • Elaboration (Appendix E) • Generation (Appendix F) • Reflection (Appendix G)	Divided among facilitators	Cognitive learning strategies worksheet (Appendix A, p. 1), large easel/flip chart with strategy already written out
15 minutes	Large-group discussion	All facilitators	Microphones
5 minutes	Workshop feedback completion	One facilitator	Commitment-to-change form (Appendix H)

Facilitators of our session included attending physician educators, nonphysician medical educators, and chief residents (although we recognize that residents, fellows, or other health care professionals could also facilitate this session). All facilitators were well versed with the book *Make It Stick: The Science of Successful Learning*.^4^ Prior to the session, we suggested that each facilitator learn about the cognitive learning strategy he or she was responsible for presenting, either through the relevant sections of the book or through other relevant materials of the facilitator's choosing. Although we had more than five facilitators available to present the workshop, two facilitators could reasonably present the session in its entirety.

The first 5 minutes of the workshop involved a brief introduction to the session's organization, goals, and objectives and to each of the facilitators (name, educational role, and hospital/program affiliation). One facilitator then gave a 15-minute interactive didactic presentation (Appendix B) that reviewed the differences between passive and active learning strategies, how active learning strategies promote learning for mastery, and how active learning strategies derive from cognitive learning theories. The presentation also included an overview of the specific cognitive learning strategies that would be practiced in small groups during the workshop.

The workshop then segued into the small-group sessions that focused on active practice of the five cognitive learning strategies over a 50-minute time frame. Participants were seated accordingly at five round tables. A limited number of chairs were assigned to each round table to encourage smaller group sizes. Groups were divided randomly, based on where participants chose to sit for the introductory portion of the workshop.

Facilitators were assigned to teach one cognitive learning strategy and rotated to each of the five tables while participants remained stationary. The facilitators were responsible for conducting a 10-minute session at each table based on their assigned strategy. Each 10-minute session was structured as follows:

• The facilitator briefly reviewed the strategy and presented what was known about it based on educational and cognitive psychology literature (2 minutes).
• The group engaged in an interactive activity to practice using the strategy and experience how it enhanced learning (5 minutes).
• The group brainstormed suggestions on how the strategy could be applied to teaching in their clinical practice settings (3 minutes).

Each of the cognitive learning strategy activities the facilitators modeled is included in Appendices C-G. Participants were also given a cognitive learning strategy worksheet on which to take notes during the small-group sessions (first page of Appendix A). Please note that for programs in which only two facilitators are available, we suggest dividing the room in half, having one facilitator present two strategies and the other facilitator present three strategies, then switching. We have found that learning from multiple presenters enlivens the activities and discussions and reduces cognitive load.

After the small-group activity concluded, all participants reconvened as a large group to reflect on the session and pose questions to the facilitators for 15 minutes. Five of the seven facilitators made up the panel, whereas the additional two facilitators walked around the audience with microphones and stimulated discussion among the large group. Participants were encouraged to share experiences, questions, and challenges related to applying these cognitive learning strategies in actual clinical teaching settings. (For programs with two facilitators, we suggest one large-group discussion.) The last 5 minutes of the workshop were reserved for a short wrap-up session during which participants were encouraged to complete the commitment-to-change assessment form (Appendix H). They were informed that they would receive an email follow-up from the facilitators in 6 weeks to inquire if they had implemented any changes to their teaching practices based on what was learned during the workshop.

### Evaluation Strategy

Our evaluation strategy was based on the commitment-to-change framework^31^ first introduced in 1982 by Purkis.^32^ The framework measures behavioral change by asking attendants of an educational session to commit to a behavioral change. The framework then keeps track of the attendants to understand their success in the implementation of the behavioral change and identifies any barriers to successful implementation. At the end of our workshop, we asked participants to voluntarily complete our commitment-to-change assessment tool (Appendix H). Six weeks after the workshop, we emailed the participants who completed the assessment tool. In the email, we included a scanned copy of their initial commitment-to-change form and asked them to complete an online assessment about their implementation successes and barriers (Appendix I). We sent reminders at 1- and 2-week intervals.

## Results

Of the 161 people registered for our workshop, 24 (14.9%) identified as residency program directors, 13 (8.1%) identified as fellowship program directors, 48 (29.8%) identified as associate residency program directors, five (3.1%) identified as associate fellowship program directors, 41 (25.5%) identified as chief or former chief residents, 10 (6.2%) identified as residents, three (1.9%) identified as hospitalists, five (3.1%) identified as coordinators, and 12 (7.4%) identified as other. We were unable to collect further data on the participants who attended the workshop and suspect that a small portion of those who registered did not attend and that a small percentage of attendants did not preregister.

**Figure 1.**
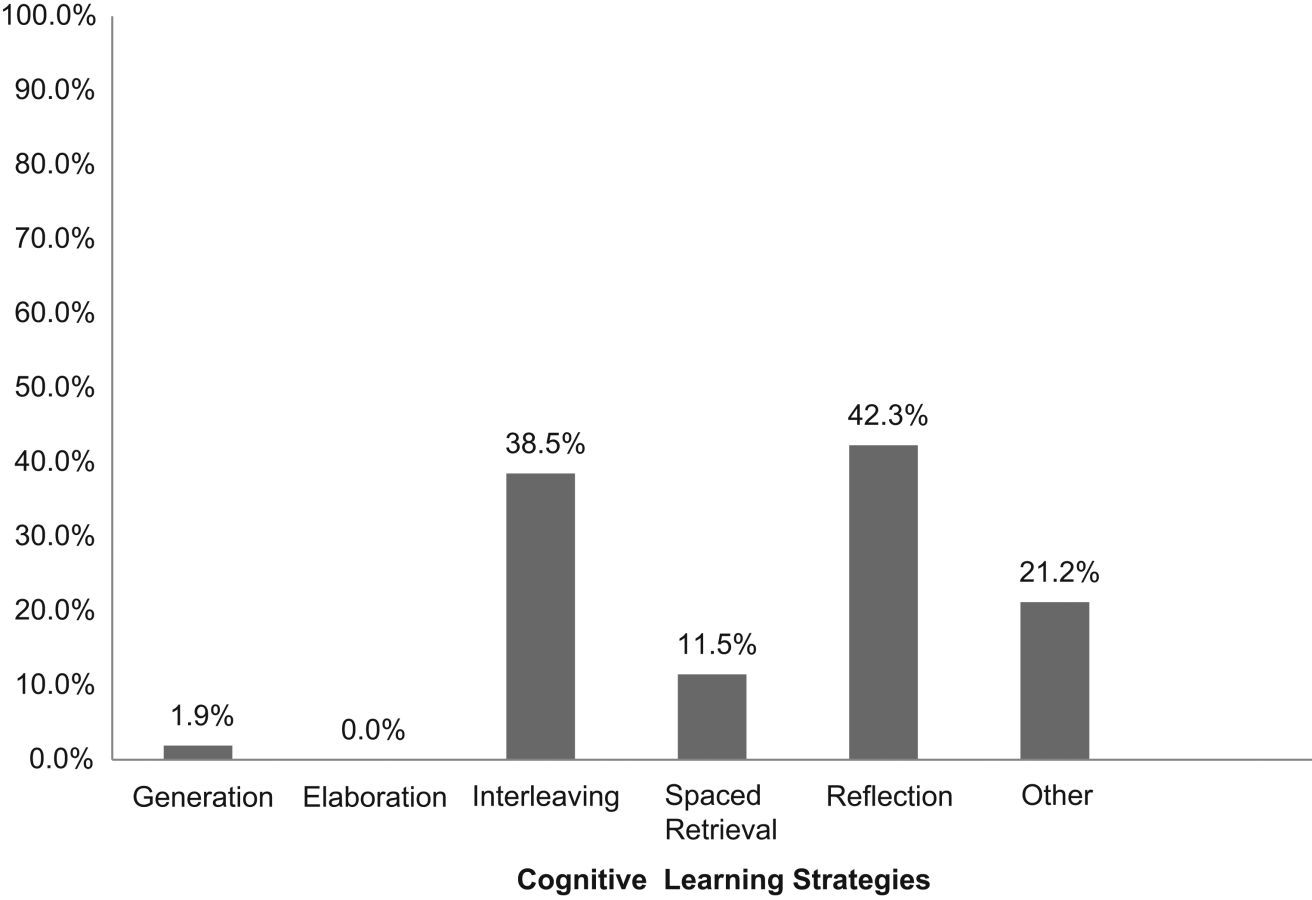
Percentage of respondents committing to each of the five cognitive learning strategies at the end of the workshop. *Other* refers to committed-to changes unrelated to the five cognitive learning strategies.

Of those who attended the workshop, 52 completed our voluntary commitment-to-change form (Appendix H) at the end of the workshop. One hundred percent of respondents reported they were planning to make a change in their teaching as a result of participating in the workshop. Two of the authors (Laura Chiel and Eli Freiman) reviewed the responses to question 2 separately and categorized the responses into the five cognitive learning strategies—spaced retrieval practice, interleaving, elaboration, generation, and reflection—and other (if unrelated or unclear relationship to a specific learning strategy). The two authors agreed on 96% of the forms. For the two forms without agreement, a third author (Lisa DelSignore) offered her categorization and prompted discussion, allowing them to reach 100% consensus. Eight respondents listed changes that corresponded to more than one cognitive learning strategy. The categorizations of these responses are listed in Figure 1. The most frequent cognitive learning strategies cited were interleaving and reflection.

**Figure 2.**
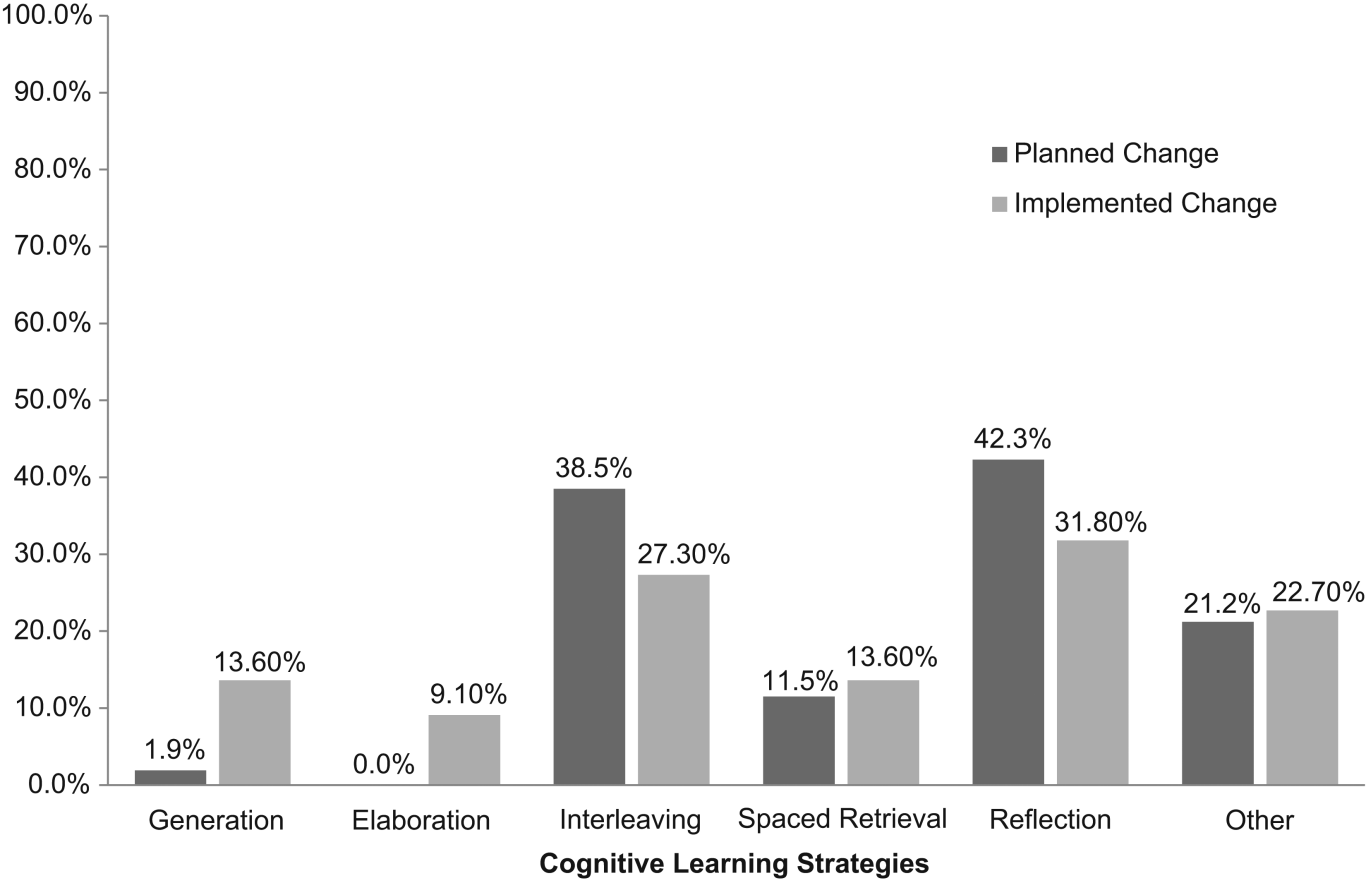
Percentage of respondents committing to each of the five cognitive learning strategies at the end of the workshop compared to percentage of respondents who implemented each of the five cognitive learning strategies at their home institutions. *Other* refers to committed-to changes unrelated to the five cognitive learning strategies.

Forty-eight of 52 respondents wrote their email addresses on the initial form (Appendix H) and were therefore able to participate in the follow-up survey. Twenty-two of these 48 participants completed the follow-up survey (Appendix I), corresponding to a 46% response rate. Eighty-two percent of respondents (*n* = 18) reported implementing a change based on the workshop. Seventy-seven percent of respondents (*n* = 17) reported implementing one of the changes they had committed to at the end of the workshop. Fifty-five percent of respondents (*n* = 12) implemented a change related to the workshop that they had not committed to at the end of the workshop. Fourteen percent of respondents (*n* = 3) said that they had not yet implemented a change. Two of the authors (Laura Chiel and Eli Freiman) again reviewed the responses to question 3 separately and categorized the responses into the five cognitive learning strategies—spaced retrieval practice, interleaving, elaboration, generation, and reflection—and other. The two authors agreed on 100% of the forms. Six respondents listed changes that corresponded to more than one cognitive learning strategy. The categorizations of these responses are shown in Figure 2. Three respondents did not implement any change based on the workshop. When asked about barriers to implementing change, two responded that they were not currently involved in any teaching, and one stated an intention to implement the change.

## Discussion

We designed a faculty development workshop to teach pediatric educators five principles of cognitive learning strategies and found that participants were able to incorporate these learning strategies into their teaching as a result of the workshop. Our evaluation strategy was based on Purkis’ commitment-to-change framework.^32^ The evaluation aligned with the third level of Kirkpatrick's evaluation model—learner behavior—in that the evaluation took place posttraining, when participants had returned to their own institutions, and determined the extent to which participants implemented or transferred what they learned during the training session.^33^ Using the commitment-to-change evaluation strategy provided solid evidence that participants gained knowledge and skills from the workshop and applied that learning to their own training programs. Those who did not implement a change reported that they either were not involved in teaching or still planned on implementing the change, without foreseeable barriers.

At the end of the workshop, participants were more committed to using interleaving and reflection than the other strategies. Interestingly, back at their home institutions, participants put all five strategies to use, suggesting that generation, elaboration, and reflection were easier to implement than participants had initially thought. In addition, more than half of the respondents reported implementing a change that they had not committed to during the workshop.

We have been able to reflect on the design and implementation of the workshop. Each activity was purposefully focused on a nonmedical topic, drawn from everyday life, allowing for presentation of the workshop across clinical disciplines and professions. Using nonmedical topics also ensured that medical information did not distract from the workshop. However, during each exercise, we brainstormed with the group how the strategy could be applied to clinical teaching. This sparked rich conversations as participants generated examples that related to their clinical settings. Participants also shared concerns about anticipated challenges and solutions to using the strategies. On further reflection, we realized that brainstorming implementation strategies could benefit from more time in future sessions. We also noted that all facilitators for the academy retreat and APPD workshop were physicians or involved in medical education. Given that these five cognitive learning strategies can be generalized to all health care professionals, it may be appropriate to have an interprofessional panel of facilitators, especially for audiences drawing from multiple professions. Inviting facilitators from varied health care professions routinely leads to the discovery of common teaching challenges and sharing of optimal solutions.

We believe that it is ideal to have five facilitators available to lead the workshop to allow one facilitator to teach each of the five cognitive learning strategies. We found that the variety of having five people rotate to each of the small groups increased the participants’ interest and eagerness to learn all of the strategies. This configuration may present challenges for those trying to replicate the workshop, as not all educational programs may have this number of available facilitators. One solution to increase the number of presenters is to include residents or fellows as facilitators, as the strategies apply to all levels of learners, regardless of rank or seniority.

The evaluation strategy we used has limitations. Only a small portion of participants participated in the commitment-to-change activity. It is possible that those who participated were more likely to incorporate change into their teaching practices than those who did not participate. Furthermore, the outcomes were self-reported, and it is possible that participants over- or underestimated their incorporation of new teaching skills.

It is our hope that educators who gain an understanding of and experience with cognitive learning strategies through our structured workshop approach will implement these strategies when they teach their learners and when they train other educators. In this way, we hope to provide tools to confront the challenge of learning and retaining vast amounts of information so that it can be recalled quickly and applied appropriately in the delivery of optimal patient care.

## Appendices

A. Handouts.docx
B. Introduction Slides.pptx
C. Spaced Retrieval Practice Facilitator Guide.docx
D. Interleaving Facilitator Guide and Handout.docx
E. Elaboration Facilitator Guide and Handout.docx
F. Generation Facilitator Guide and Handout.docx
G. Reflection Facilitator Guide and Handout.docx
H. Commitment-to-Change Initial Form.docx
I. Commitment-to-Change Follow-up Form.docx All appendices are peer reviewed as integral parts of the Original Publication.
