# Supplementary material for: Applying Cognitive Learning Strategies to Enhance Learning and Retention in Clinical Teaching Settings
Source: MedEdPORTAL. 2019 Nov 1;15:10850. doi: 10.15766/mep_2374-8265.10850 (PMC6946583; doi:10.15766/mep_2374-8265.10850)
Supplement: Supplementary file 1 — A. Handouts.docx B. Introduction Slides.pptx C. Spaced Retrieval Practice Facilitator Guide.docx D. Interleaving Facilitator Guide and Handout.docx E. Elaboration Facilitator Guide and Handout.docx F. Generation Facilitator Guide and Handout.docx G. Reflection Facilitator Guide and Handout.docx H. Commitment-to-Change Initial Form.docx I. Commitment-to-Change Follow-up Form.docx [file mep-15-10850-s001.zip › A. Handouts.docx]

| **Learning Strategies** | **Notes** |
| --- | --- |
| **Spaced Retrieval Practice**  Studying information more than once but leaving considerable time between practice sessions |  |
| **Elaboration**  Giving new information meaning by expressing it in your own words and connecting it with what you already know |  |
| **Interleaving**  Learning more than one concept at a time so that you can alternate between different problems that call for different solutions |  |
| **Generation**  Trying to answer a question or solve a problem before being presented any cues, information, or solutions |  |
| **Reflection**  Reviewing what has just been learned as a tool for consolidating knowledge |  |

Spaced Retrieval Practice Handout:

| Acronym | What do you think this stands for? |
| --- | --- |
| DOB |  |
| OTC |  |
| RAM |  |
| EST |  |
| SSN |  |
| SUV |  |
| RACE |  |
| AARP |  |

Interleaving Practice Handout (page 1):

African Elephant

Image by Matt Artz, retrieved from <https://images.unsplash.com/photo-1503889678302-211ae988b095?ixlib=rb-0.3.5&ixid=eyJhcHBfaWQiOjEyMDd9&s=7ce46670122460d71fac08ed99a6aec6&auto=format&fit=crop&w=1952&q=80> on 10/24/18. Creative Commons License associated: https://creativecommons.org/publicdomain/zero/1.0/

Asian Elephant

Image by Saray Jimenez, retrieved from <https://images.unsplash.com/photo-1513863525216-babdb36af01c?ixlib=rb-0.3.5&ixid=eyJhcHBfaWQiOjEyMDd9&s=b6c59f9152229d2ab3514fb598dad594&auto=format&fit=crop&w=3140&q=80> on 10/24/18. Creative Commons License associated: https://creativecommons.org/publicdomain/zero/1.0/

Interleaving Practice Handout (page 2):

Forest Elephant


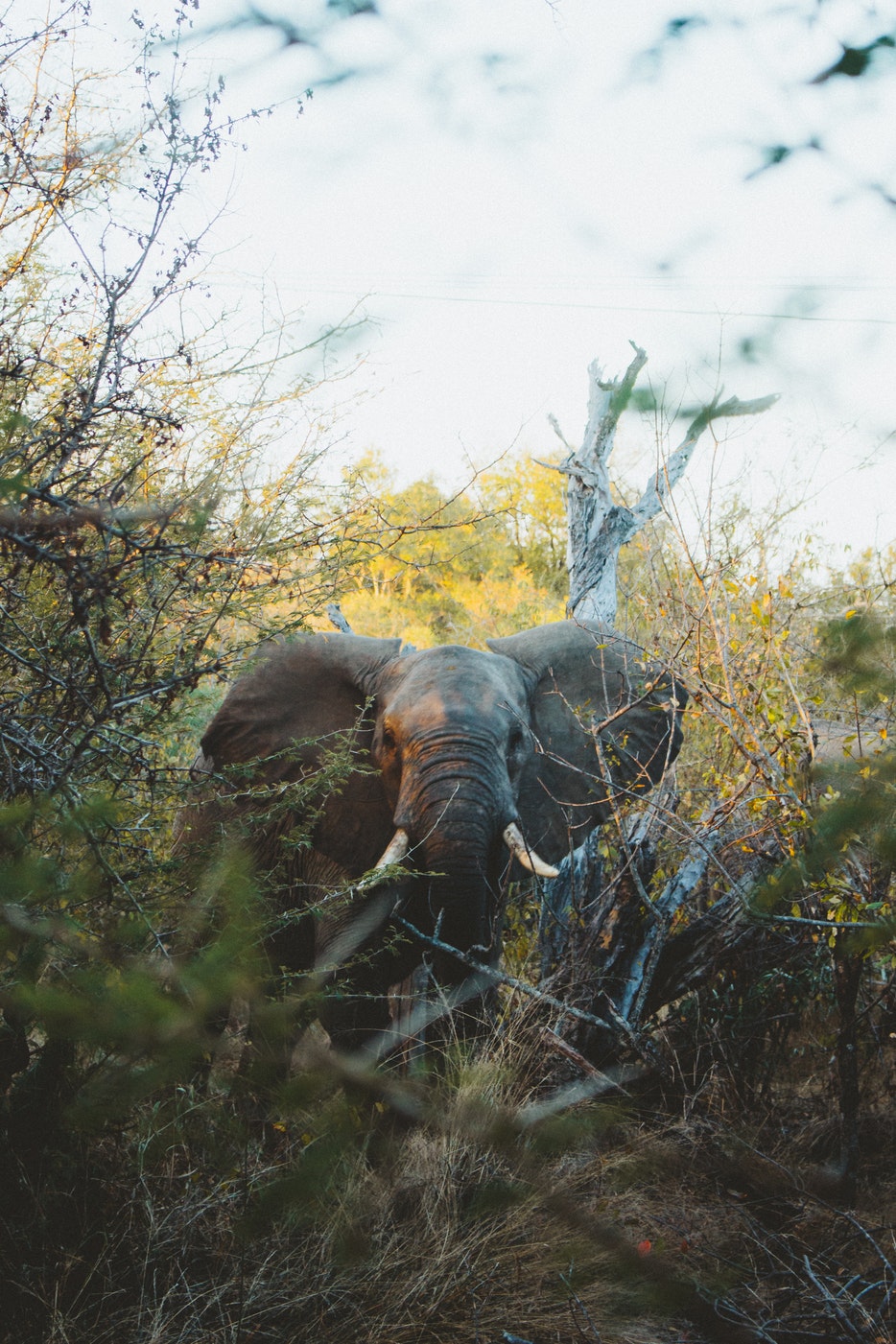


Image by Graham Hunt, retrieved from <https://images.unsplash.com/photo-1516786549992-7fc60748b3a3?ixlib=rb-0.3.5&ixid=eyJhcHBfaWQiOjEyMDd9&s=bfd032206cc2c3825c7284be692c8fc4&auto=format&fit=crop&w=934&q=80> on 10/24/18. Creative Commons License associated: https://creativecommons.org/publicdomain/zero/1.0/

Elaboration Practice Handout:

***Elaboration is the process of giving new information meaning by connecting it with what you already know and expressing it in your own words.***

What are the three methods of heat transfer? What are some examples?

Brainstorming activity: How might you use the concept of elaboration in your own teaching or clinical setting?

Generation Practice Handout (page 1):

Generation Practice Handout (page 2):

Purine Catabolism

Hypoxanthine

Xanthine

Uric Acid

Allantoin

**BAD**

XO

XO

UO

Rasburicase

Allopurinol

Reflection Practice Handout:

Reflection Using the Four-Question Technique^1^

1. Identify one important concept, research finding, theory, or idea that you learned while completing this activity. (analyzing)
2. Why do you believe that this concept, research finding, theory, or idea is important? (reflecting)
3. Apply what you have learned from this activity to some aspect of your life. (relating)

4.    What question(s) has the activity raised for you?  What are you still wondering about? (questioning).

**References:**

1. Dietz-Uhler B, Lanter JR. Using the four-questions technique to enhance learning. Teach Psychol. 2009;36(1):38-41.
