# Supplementary material for: Applying Cognitive Learning Strategies to Enhance Learning and Retention in Clinical Teaching Settings
Source: MedEdPORTAL. 2019 Nov 1;15:10850. doi: 10.15766/mep_2374-8265.10850 (PMC6946583; doi:10.15766/mep_2374-8265.10850)
Supplement: Supplementary file 1 — A. Handouts.docx B. Introduction Slides.pptx C. Spaced Retrieval Practice Facilitator Guide.docx D. Interleaving Facilitator Guide and Handout.docx E. Elaboration Facilitator Guide and Handout.docx F. Generation Facilitator Guide and Handout.docx G. Reflection Facilitator Guide and Handout.docx H. Commitment-to-Change Initial Form.docx I. Commitment-to-Change Follow-up Form.docx [file mep-15-10850-s001.zip › B. Introduction Slides.pptx]

## Slide 1
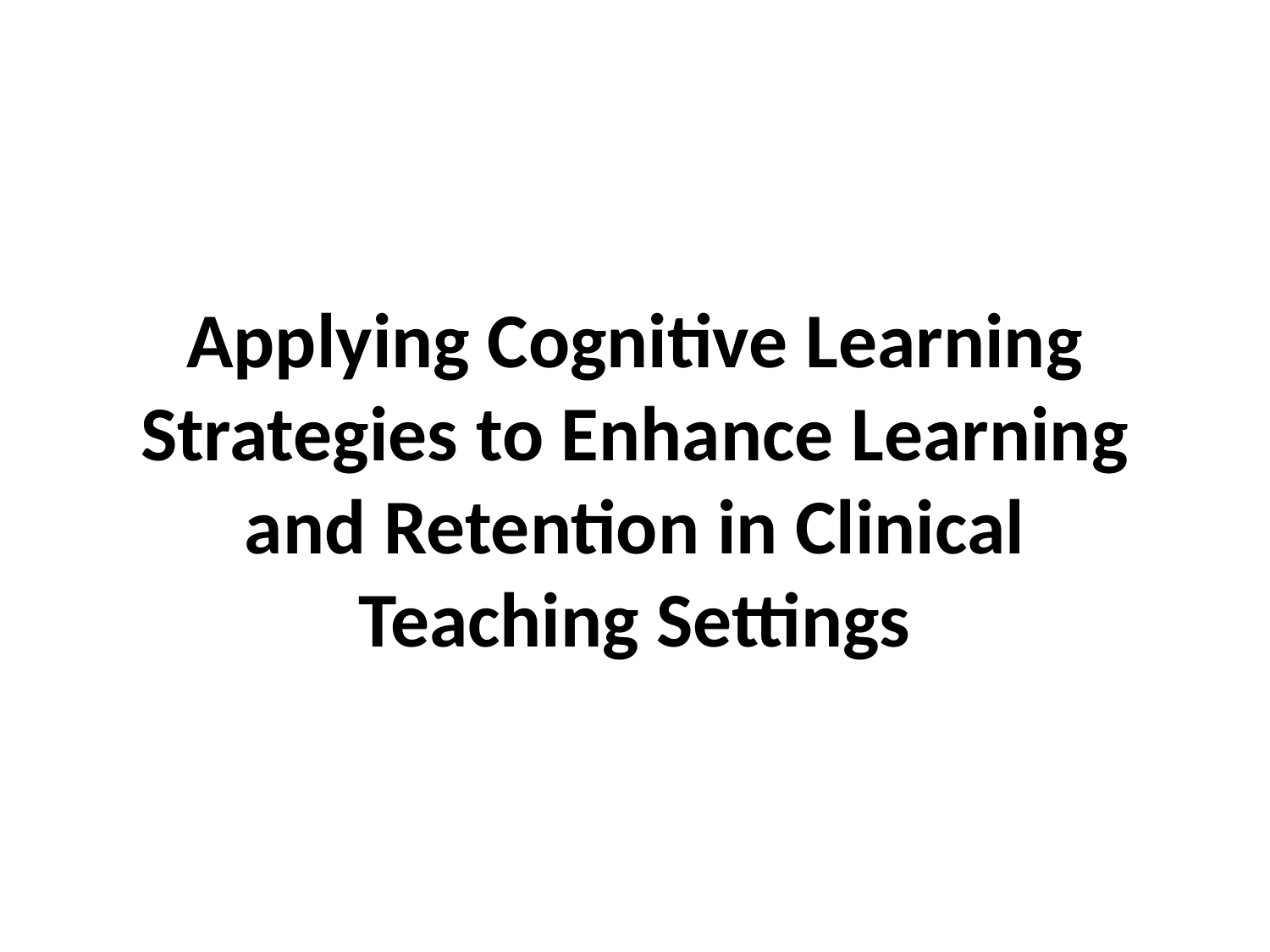

# Applying Cognitive Learning Strategies to Enhance Learning and Retention in Clinical Teaching Settings

## Slide 2
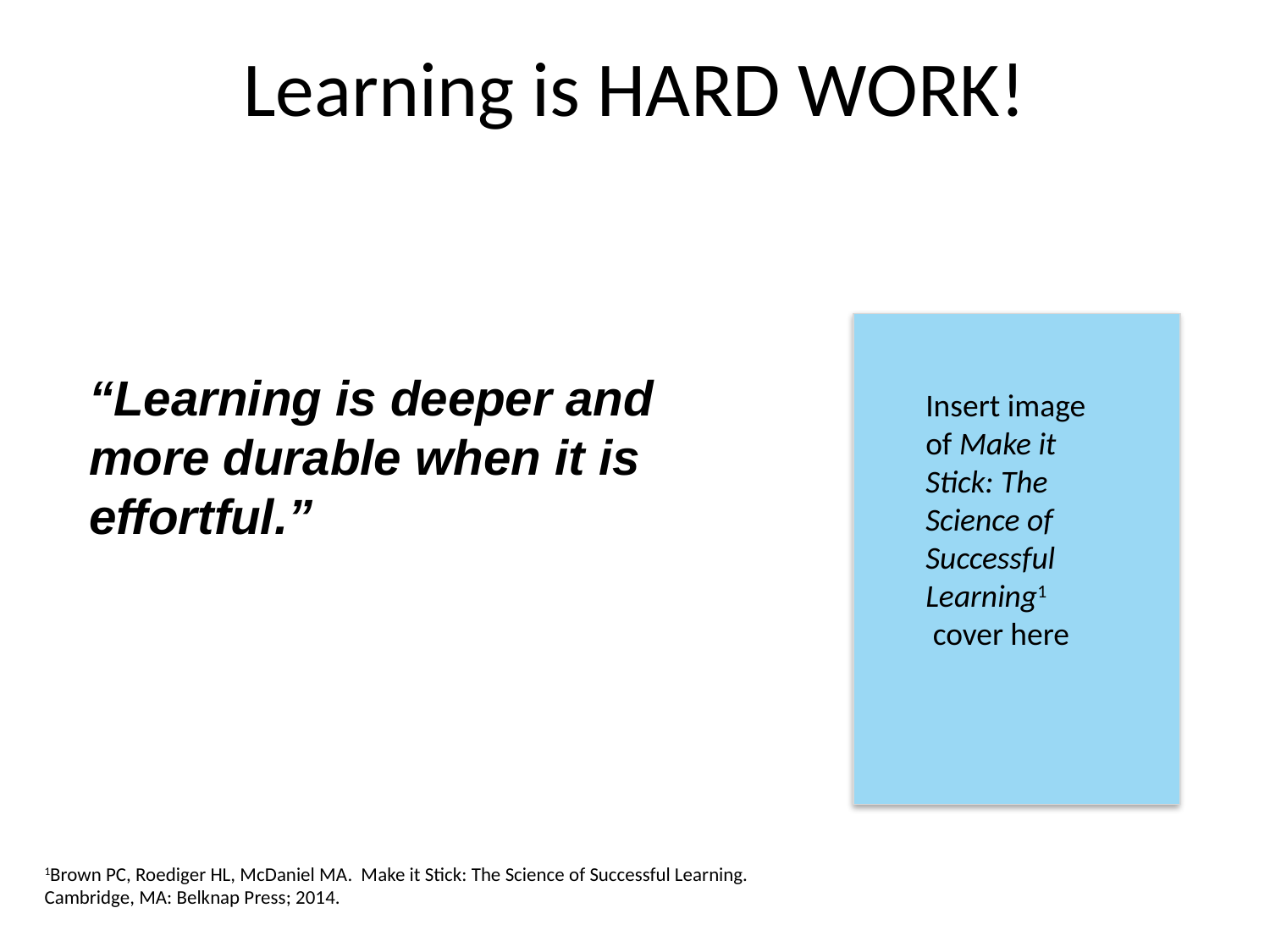

# Learning is HARD WORK!
“Learning is deeper and more durable when it is effortful.”
Insert image of Make it Stick: The Science of Successful Learning1
 cover here
1Brown PC, Roediger HL, McDaniel MA. Make it Stick: The Science of Successful Learning. Cambridge, MA: Belknap Press; 2014.

## Slide 3
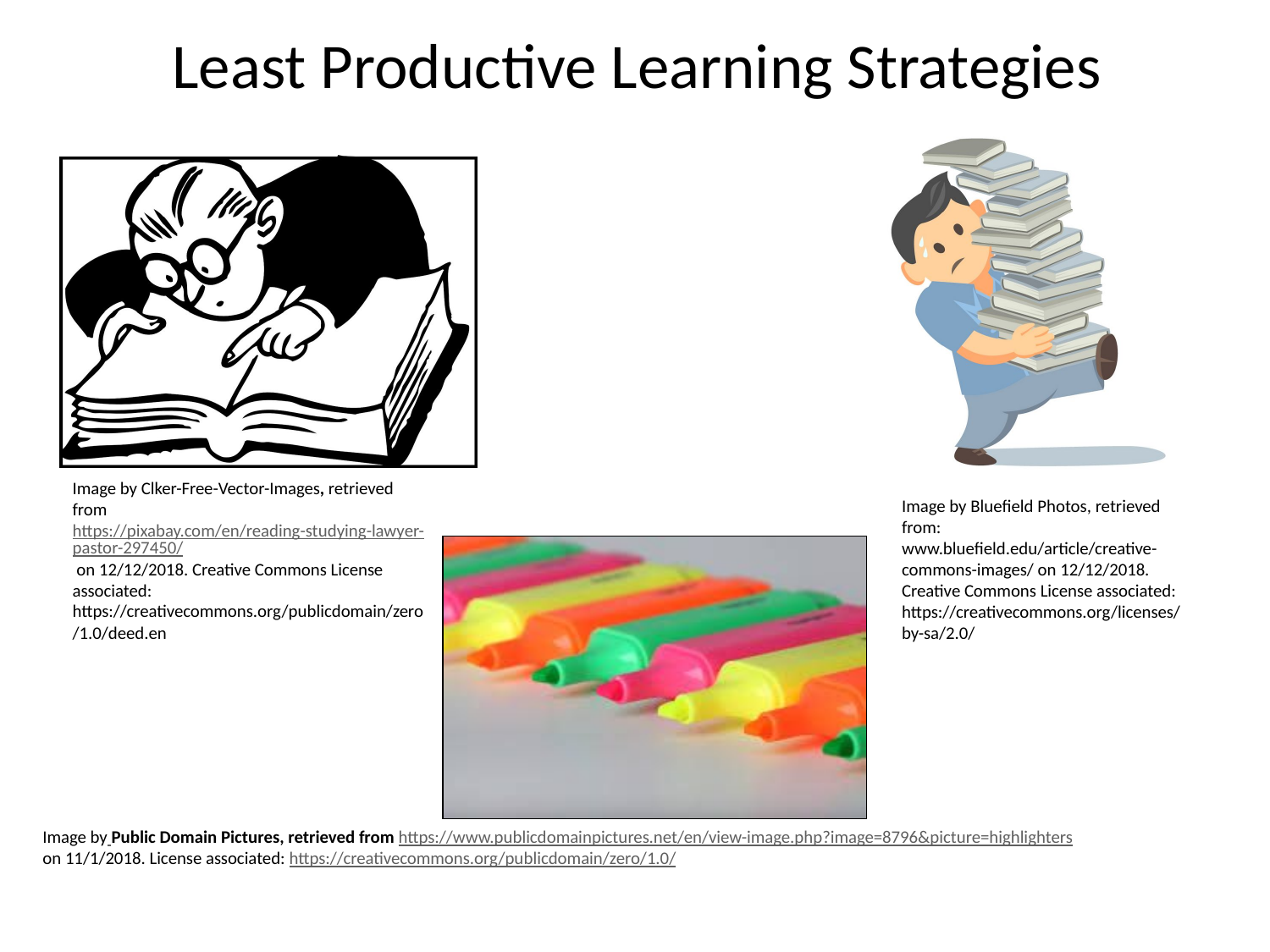

# Least Productive Learning Strategies
Image by Bluefield Photos, retrieved from:
www.bluefield.edu/article/creative-commons-images/ on 12/12/2018. Creative Commons License associated: https://creativecommons.org/licenses/by-sa/2.0/
Image by Clker-Free-Vector-Images, retrieved from https://pixabay.com/en/reading-studying-lawyer-pastor-297450/ on 12/12/2018. Creative Commons License associated: https://creativecommons.org/publicdomain/zero/1.0/deed.en
Image by Public Domain Pictures, retrieved from https://www.publicdomainpictures.net/en/view-image.php?image=8796&picture=highlighters
on 11/1/2018. License associated: https://creativecommons.org/publicdomain/zero/1.0/

## Slide 4
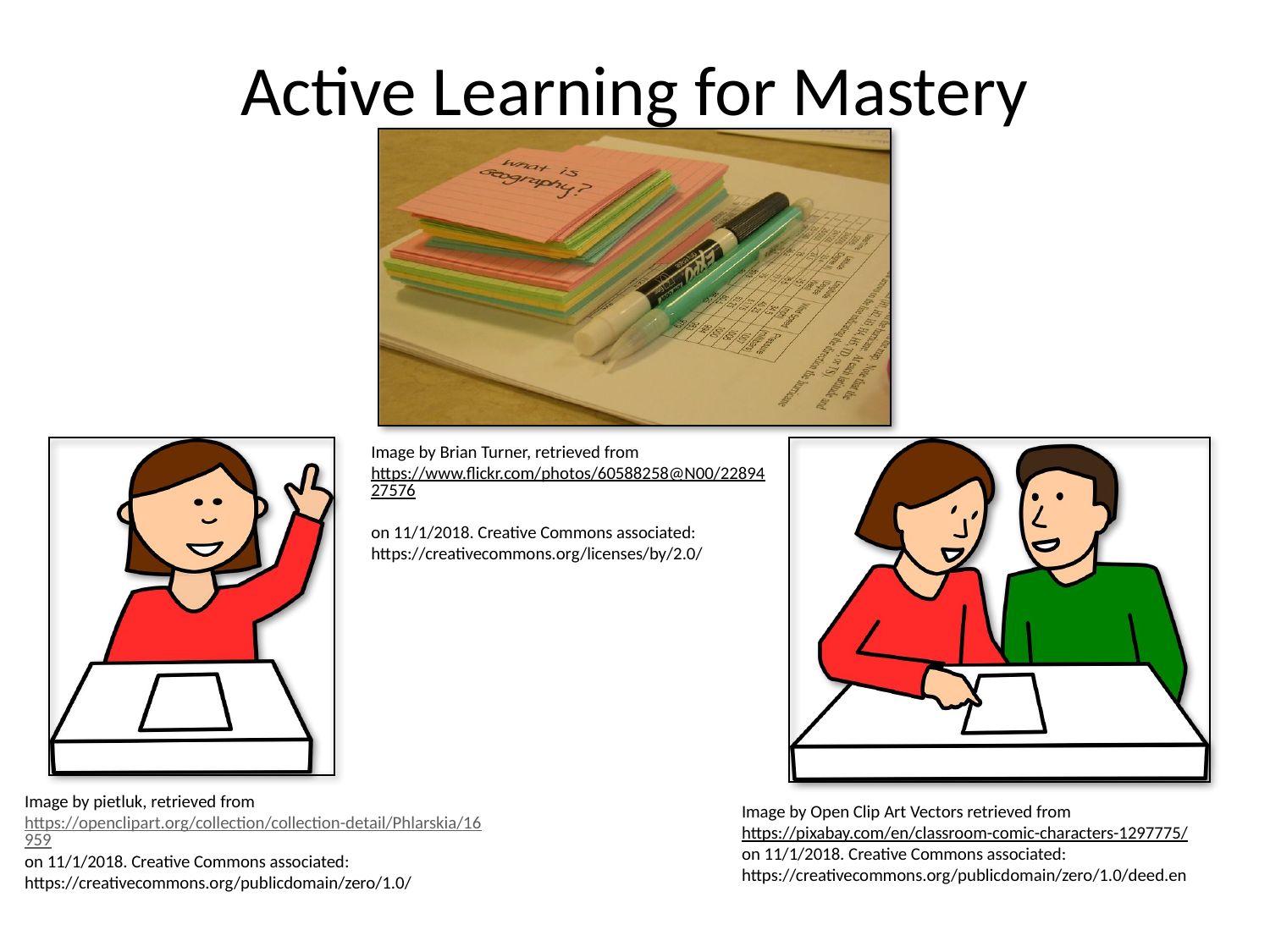

# Active Learning for Mastery
Image by Brian Turner, retrieved from
https://www.flickr.com/photos/60588258@N00/2289427576
on 11/1/2018. Creative Commons associated: https://creativecommons.org/licenses/by/2.0/
Image by pietluk, retrieved from https://openclipart.org/collection/collection-detail/Phlarskia/16959
on 11/1/2018. Creative Commons associated: https://creativecommons.org/publicdomain/zero/1.0/
Image by Open Clip Art Vectors retrieved from
https://pixabay.com/en/classroom-comic-characters-1297775/
on 11/1/2018. Creative Commons associated: https://creativecommons.org/publicdomain/zero/1.0/deed.en

## Slide 5
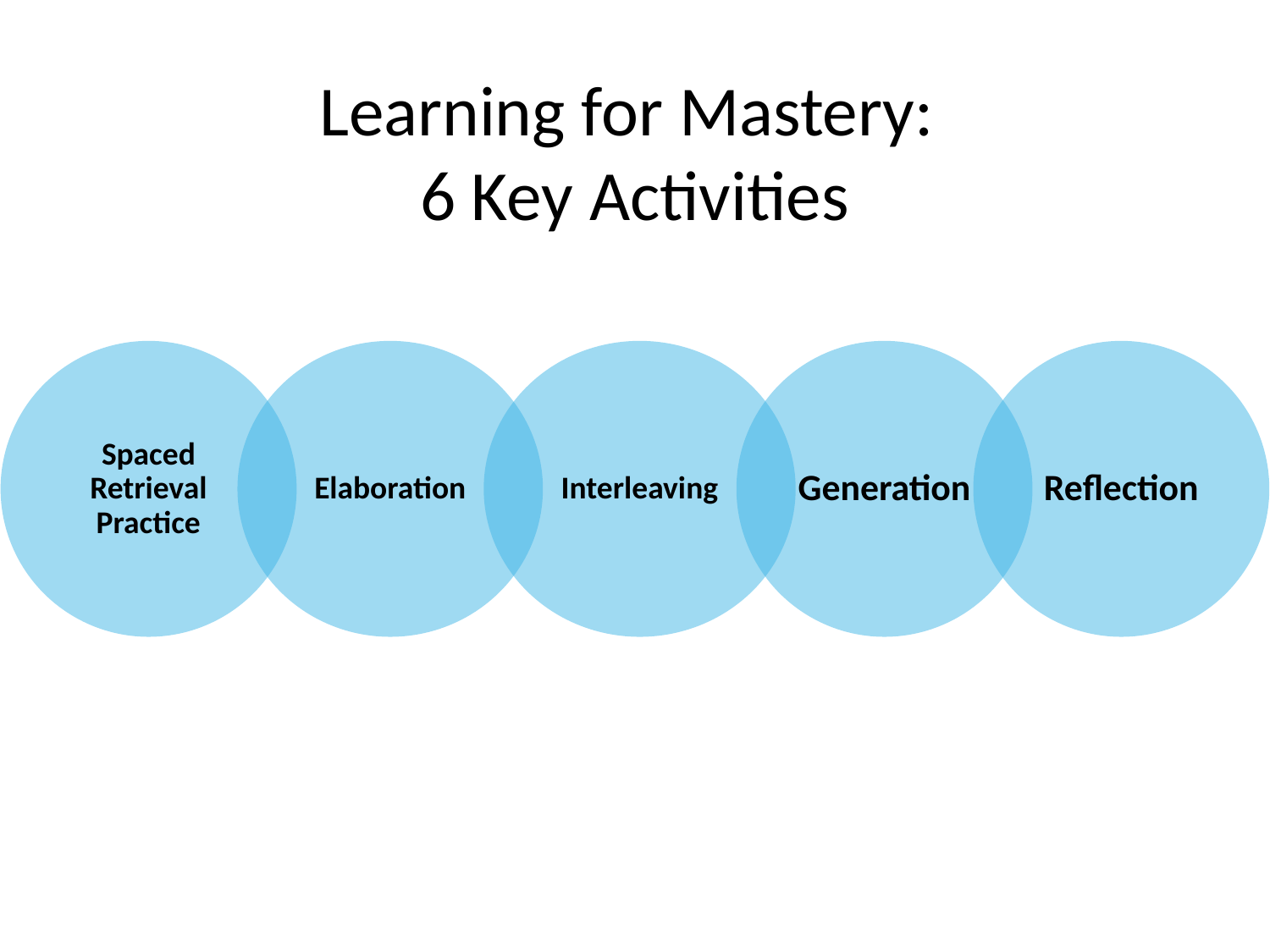

# Learning for Mastery: 6 Key Activities

## Slide 6
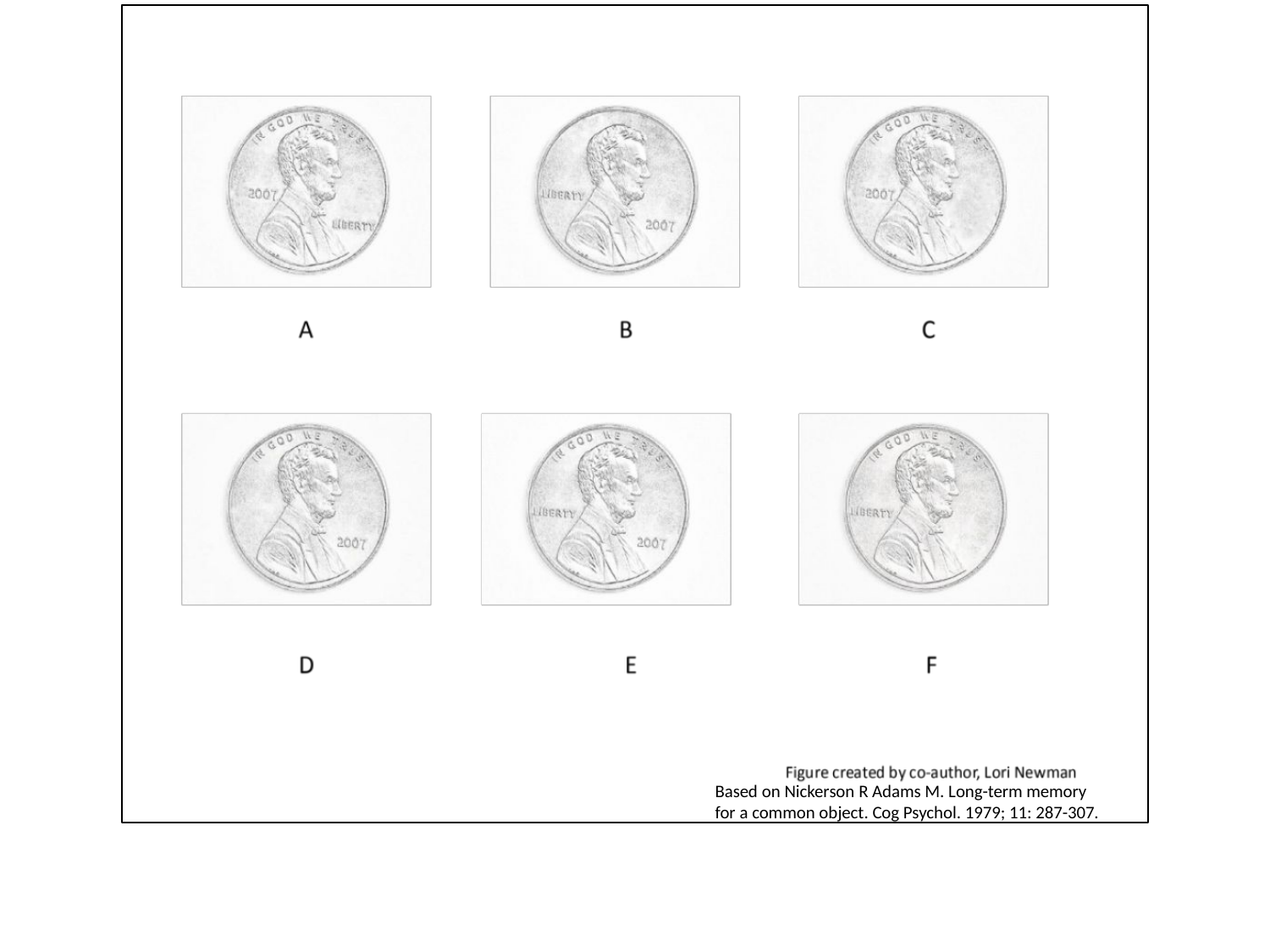

Based on Nickerson R Adams M. Long-term memory for a common object. Cog Psychol. 1979; 11: 287-307.

## Slide 7
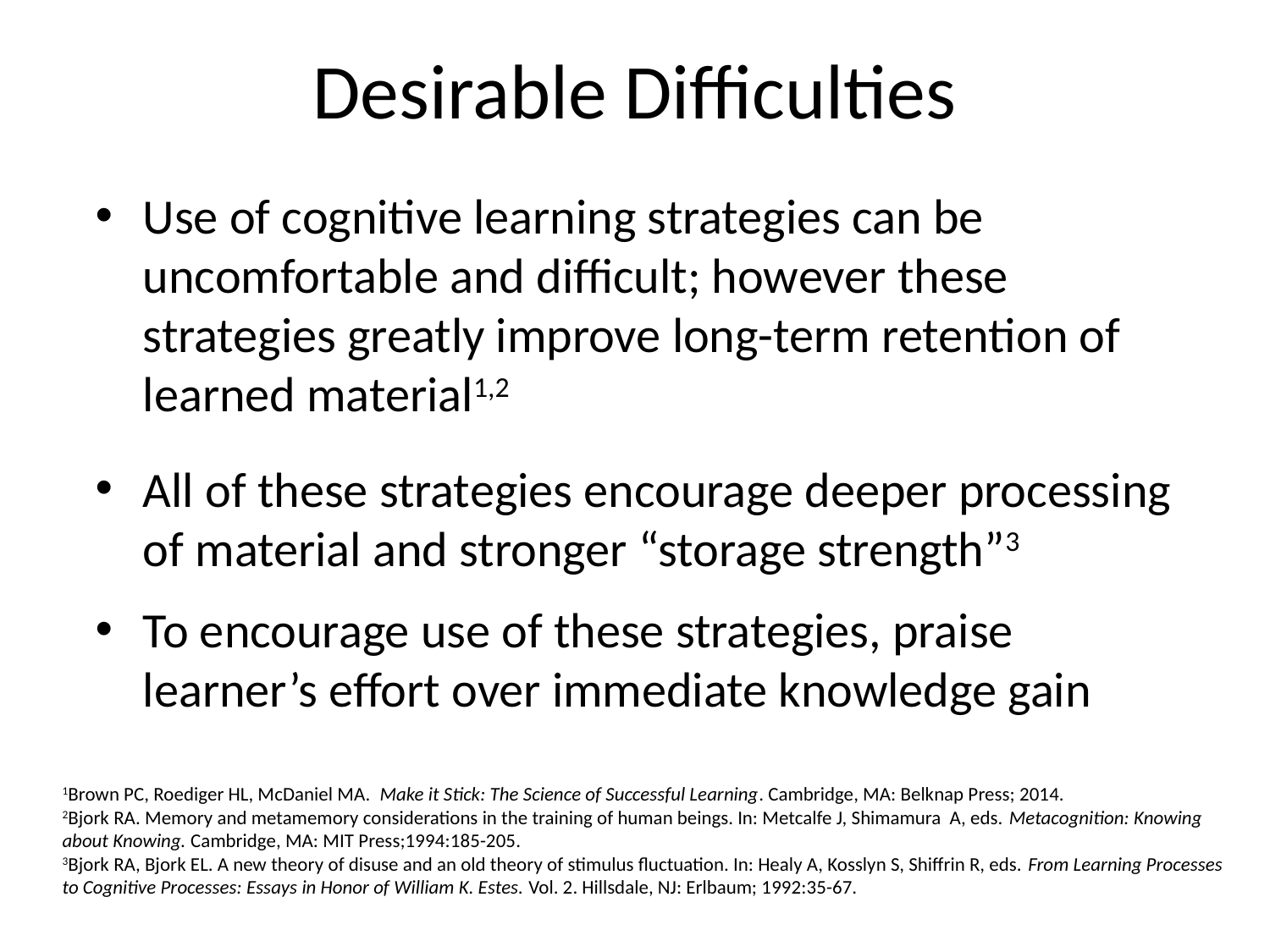

# Desirable Difficulties
Use of cognitive learning strategies can be uncomfortable and difficult; however these strategies greatly improve long-term retention of learned material1,2
All of these strategies encourage deeper processing of material and stronger “storage strength”3
To encourage use of these strategies, praise learner’s effort over immediate knowledge gain
1Brown PC, Roediger HL, McDaniel MA. Make it Stick: The Science of Successful Learning. Cambridge, MA: Belknap Press; 2014.
2Bjork RA. Memory and metamemory considerations in the training of human beings. In: Metcalfe J, Shimamura A, eds. Metacognition: Knowing about Knowing. Cambridge, MA: MIT Press;1994:185-205.
3Bjork RA, Bjork EL. A new theory of disuse and an old theory of stimulus fluctuation. In: Healy A, Kosslyn S, Shiffrin R, eds. From Learning Processes to Cognitive Processes: Essays in Honor of William K. Estes. Vol. 2. Hillsdale, NJ: Erlbaum; 1992:35-67.

## Slide 8
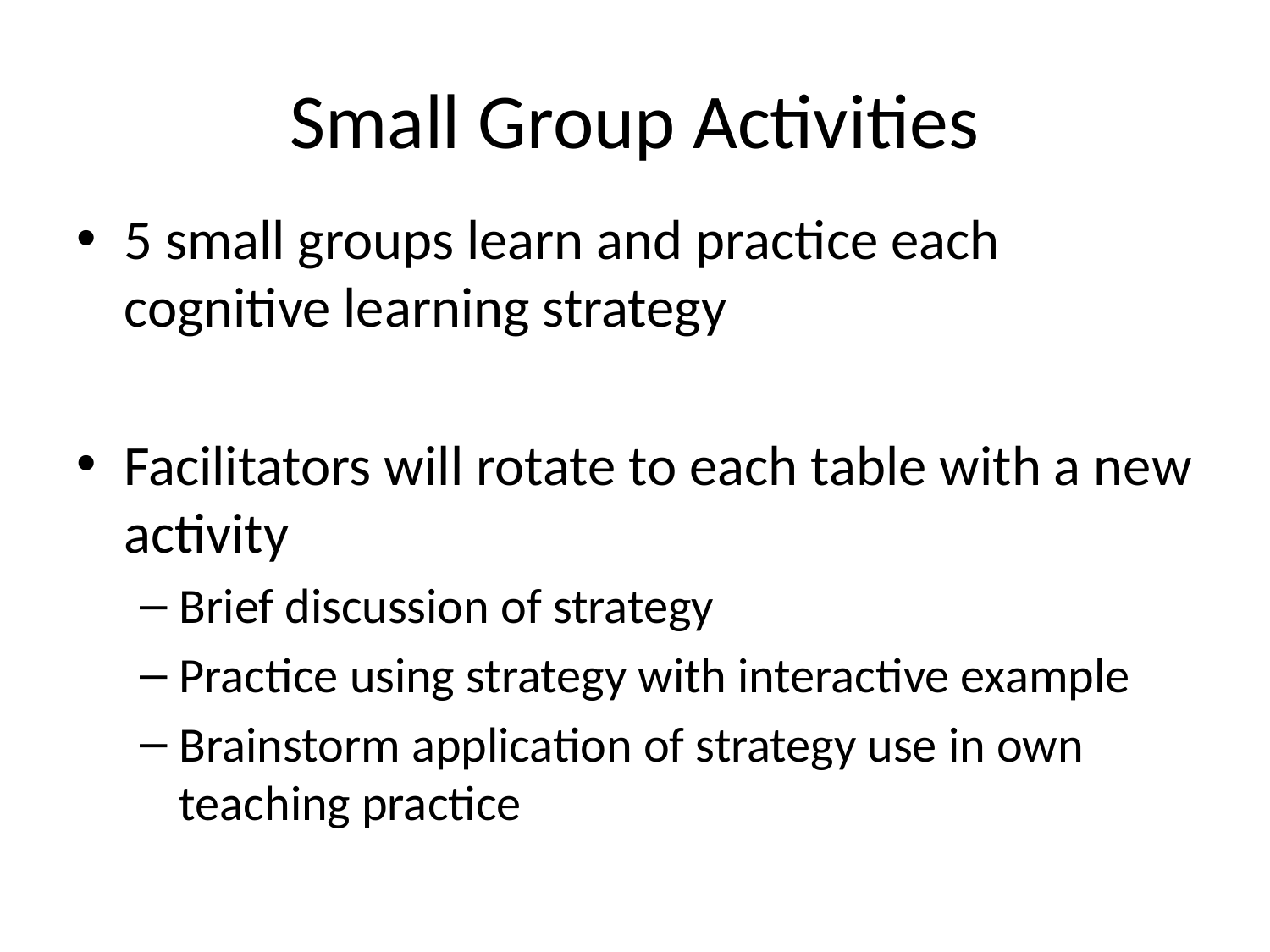

# Small Group Activities
5 small groups learn and practice each cognitive learning strategy
Facilitators will rotate to each table with a new activity
Brief discussion of strategy
Practice using strategy with interactive example
Brainstorm application of strategy use in own teaching practice
8
