# Supplementary material for: Applying Cognitive Learning Strategies to Enhance Learning and Retention in Clinical Teaching Settings
Source: MedEdPORTAL. 2019 Nov 1;15:10850. doi: 10.15766/mep_2374-8265.10850 (PMC6946583; doi:10.15766/mep_2374-8265.10850)
Supplement: Supplementary file 1 — A. Handouts.docx B. Introduction Slides.pptx C. Spaced Retrieval Practice Facilitator Guide.docx D. Interleaving Facilitator Guide and Handout.docx E. Elaboration Facilitator Guide and Handout.docx F. Generation Facilitator Guide and Handout.docx G. Reflection Facilitator Guide and Handout.docx H. Commitment-to-Change Initial Form.docx I. Commitment-to-Change Follow-up Form.docx [file mep-15-10850-s001.zip › C. Spaced Retrieval Practice Facilitator Guide.docx]

**Spaced Retrieval Practice Small Group Session**

User Guide:

- Suggested facilitator wording is noted in quotations

- Instructions are noted in italics

Background on Principle (*2 minutes)*:

“Spaced retrieval practice is the understanding that is better to recall learned information at scheduled (and increasingly longer) intervals in order to enhance retainment of that information. This practice leads to greater knowledge gain and retention as compared to massed practice, where the learner packs a wealth of information in during a single session and does not review it again. The reasoning behind why spaced retrieval practice leads to greater knowledge gain and enhanced memory retention is because it takes time to strengthen the cognitive pathways involved in building meaning of newly learned information and to connect this information with pre-existing knowledge. Once this process is complete, the information becomes consolidated in long-term memory. The effort of then retrieving this information from memory after a spaced interval of time strengthens the level of cognitive processing. Effortful learning in this way leads to mastery of content, but may take hours, days, weeks, or months to achieve based on the complexity of the concept and the learner level.”^1-5^

“The benefits for spacing out practice are long established, but for a relevant example, consider a study of 38 surgical residents who took part in a series of 4 short lessons in microsurgery. Each lesson included some instruction followed by some practice. Half the trainees completed all 4 lessons in a single day. The others completed the same 4 lessons but with a week’s interval between them. When they were evaluated a month after their last session, those who had lessons spaced a week apart outperformed their colleagues in all areas – elapsed time to complete a surgery, number of hand movement, and success at reattaching the severed aorta of live rats. The difference in performance between the 2 groups was significant; the residents who had taken all 4 sessions massed in a single day not only scored lower on all measures, but 16% damaged the vessels beyond repair and were unable to complete their surgeries.”^6^

Activity *(3 minutes):*

*Hand out page 3*. “Now I am going to have you experience ‘retrieval practice.’ Take some time to review the handout, which has a list of acronyms on it. Write down what you think they stand for.

**Correct Answers**

*DOB (Date of Birth)*

*OTC (Over the Counter)*

*RAM (Random Access Memory)*

*EST (Eastern Standard Time)*

*SSN (Social Security Number)*

*SUV (Sports Utility Vehicle)*

*RACE (Rescue, Alarm, Close, Evacuate)*

*AARP (American Association of Retired Persons)*

*Give them 1-2 minutes to think about their responses and then address the group:*

“Ok, now let’s see how you did.”

*Proceed to say each acronym out loud and ask the group for their proposed response, validating or correcting each response, as appropriate. This should take 1 minute to review.*

“My guess is you didn’t get the ones that you don’t use on a regularly spaced interval, even if you’ve been exposed to them previously. For example, all of you knew ‘DOB’ was date of birth because at regular intervals you are asked for this information on many forms. This is in contrast to ‘RACE,’ which you are exposed to on a yearly basis through fire safety training, but don’t use on a regular basis, and, as such, have a harder time retrieving the meaning from memory.

Given that true “spacing” practice requires scheduled *future* time intervals by definition, today’s activity does not embody the full definition of spaced practice. However the idea is that you have presumably had some previous exposure to these acronyms so that the time point of this activity is in fact a ‘spaced’ time point. If you continue to review this list weekly for the next 4 weeks and then test yourself 1 week later, you will most likely find you have a greater chance at retrieving what all the acronyms stand for.”

Brainstorm activity of how the group can use this skill in their teaching settings (*5 minutes*):

“Now I’d like everyone to think about how you might use the concept of spaced retrieval practice in your own teaching setting and have people share their thoughts with the group. I would also be happy to take any questions about spaced retrieval practice at this time.”

*Try to hear at least 2 suggestions before you move on to the next group.*

**References:**

1. Pashler H, Rohrer D, Cepeda NJ, Carpenter SK. Enhancing learning and retarding forgetting: choices and consequences. *Psycho Bull Rev.* 2007; 14(2): 187-193.
2. Agarwal PK, Roediger HL, McDaniel MA, McDermott KB. How to Use Retrieval Practice to Improve Learning. St. Louis, WA: Washington University St. Louis; 2018. <http://pdf.retrievalpractice.org/RetrievalPracticeGuide.pdf> Accessed May 12, 2019.
3. Kerfoot BP, Shaffer K, McMahon GT, Baker H, Kirdar J, Kanter S, et al. Online “spaced education progress testing” of students to confront two upcoming challenges to medical schools. *Acad Med*. 2011; 86(3):300-306.
4. Kerfoot BP, Baker H, Pangaro L, Agarwal K, Taffet G, Mechaber AJ, Armstrong EG. An online spaced-education game to teach and assess medical students: a multi-institutional prospective trial. *Acad Med*. 2012; 87: 1443-1447.
5. Kerfoot BP, DeWolf WC, Masser BA, Church PA, Federman DD. Spaced education improves the retention of clinical knowledge by medical students: a randomized controlled trial. *Med Educ*. 2007;41(1):23-31.
6. Moulton CA, Dubrowski A, Macrae H, Graham B, Grober E, Reznick R. Teaching surgical skills: what kind of practice makes perfect? A randomized, controlled trial. *Ann Surg*. 2006;244(3):400-409.

Spaced Retrieval Practice Handout:

| Acronym | What do you think this stands for? |
| --- | --- |
| DOB |  |
| OTC |  |
| RAM |  |
| EST |  |
| SSN |  |
| SUV |  |
| RACE |  |
| AARP |  |
