# Supplementary material for: Applying Cognitive Learning Strategies to Enhance Learning and Retention in Clinical Teaching Settings
Source: MedEdPORTAL. 2019 Nov 1;15:10850. doi: 10.15766/mep_2374-8265.10850 (PMC6946583; doi:10.15766/mep_2374-8265.10850)
Supplement: Supplementary file 1 — A. Handouts.docx B. Introduction Slides.pptx C. Spaced Retrieval Practice Facilitator Guide.docx D. Interleaving Facilitator Guide and Handout.docx E. Elaboration Facilitator Guide and Handout.docx F. Generation Facilitator Guide and Handout.docx G. Reflection Facilitator Guide and Handout.docx H. Commitment-to-Change Initial Form.docx I. Commitment-to-Change Follow-up Form.docx [file mep-15-10850-s001.zip › D. Interleaving Facilitator Guide and Handout.docx]

**Interleaving Small Group Session:**

User Guide:

- Suggested facilitator wording is noted in quotations

- Instructions are noted in italics

Background on Principle (*2 minutes)*:

“Interleaving is a process of alternating between types of information — whether it is subject areas, topics, or clinical diseases — rather than presenting them in an isolated or linear fashion. The purpose is to compare types of information, which improves learning, rather than trying to absorb a mass of information in one block of time. Think of how children are taught math. They study addition for a week, next they study subtraction, then multiplication, etc. This is ‘mass practice’ repeatedly studying the same thing before moving on to the next content area. With Interleaving, subject areas are mixed to enhance learning, a little addition, a little subtraction, etc. This not only leads to longer term retention, but also allows the learner to make associations, discriminate, and understand concepts.^1-4^

Think about using Interleaving in the study of art: the first group of learners studies examples of one painter’s works and then moves onto studying another painter’s work, learning the styles of each and focusing on the commonalities in the work of the individual. Another group studies the works of multiple painters at the same time, moving back and forth, learning the styles of each by understanding the differences. This takes learning to a higher level of comprehension.^1-5^

Interleaving is beneficial due to the act of contrasting different problem types. This requires the learner to make a judgment or choose from different strategies to solve each problem. The idea is that such practice encourages additional organizational processing to identify critical features between problem types. This leads to stronger understanding of a concept or skillbeing practiced.^1^”

Activity (*5 minutes*):

“Now we’ll have a brief experiment with Interleaving by looking at elephants. There are two main kinds of elephants, African elephants (or African savanna elephants) and Asian elephants. We think of them as two types of the same animal, but they are actually two different species. African elephants (*Loxodonta africana*) and Asian elephants (*Elephas maximus*) are both descended from mammoths, but diverged a long time ago and cannot interbreed. They have many similarities, but they also have many differences. By examining their characteristics, we can think about how these differences may have developed and work to identify animals that may be related.”

*Distribute the handout (page 3) with the two different elephants.*

“Look at these pictures and tell me what you see? What is similar and what is different between these two elephants? *Allow the group to make their observations. The most significant differences are below:*

- African elephants have larger ears, Asian elephants have smaller ears. We can speculate why by thinking about their habitat and the African elephant’s need to cool themselves by fanning their ears. The African elephant’s ears also look like the continent of Africa.
- Head shape also differentiates the two with African elephants having round domed heads and Asian elephants having a “twin dome.”
- In this photo, the African elephant has tusks and the Asian elephant does not. You might think this is because the Asian elephant is female, but in fact, so is the African elephant. All African elephants have tusks, both male and female, but only male Asian elephants have tusks.
- Another difference that is more subtle is the feet of these elephants. You can’t see it well in these pictures, but African elephant has 4 or 5 toenails on the front feet and 3 to 4 toenails on the hind feet. Asian elephant has 5 toenails on the front feet and 4 to 5 toenails on the hind feet.
- While they may look similar, the trunks of these elephants are different. The African elephants have two “fingers” on the end of their trunk to grip things while the Asian elephant has one “finger” to curl around food, which they then squeeze up into their mouths.

“So now we’re going to look at a third type of elephant, the forest elephant.” *Distribute page 4.*

“Based on what you learned about elephants, which of the elephant species is the forest elephant more closely related?” *Let the group discuss.* “The forest elephant is more closely related to the African savanna elephant. It lives in Africa and its scientific name is similar (*Loxodonta cyclotis*). You might have made your decision because of the ears and head in this picture. The ears are large and shaped similarly to the African elephant and the head has a single dome. This elephant also has tusks, but we don’t know if it is male or female, so that doesn’t distinguish it.”

“What have we learned, besides some interesting things about elephants? We could just have looked at one elephant, examining its features and then turn to look at the other in a similar manner. However, with Interleaving, we examined the seemingly similar features and highlighted the differences between the two species, making it easier to remember the features typical of each and to think about why these differences may occur. It also allowed us to apply that knowledge to a new situation and identify yet another type of elephant.”

Brainstorm activity of how the group can use this skill in their teaching settings (3 minutes):

“Now I’d like everyone to think about how you might use the concept of Interleaving in your own teaching setting.”

*Try to hear as many suggestions as time allows.*

References:

1. Bryck R. Mix it up to make it stick: what research says about “Interleaving.” Landmark College Research and Training Blog. February 2019. <https://www.landmark.edu/research-training/blog/making-learning-stick-research-on-interleaving> Accessed May 12, 2019.
2. Dunlosky J, Rawson KA, Marsh EJ, Nathan MJ, Willingham DT. Improving students' learning with effective learning techniques: promising directions from cognitive and educational psychology. *Psychol Sci Public Interest*. 2013;14:4–58.
3. Gooding HC, Mann K, Armstrong E. Twelve tips for applying the science of learning to health professions education. *Med Teach*. 2017;39(1):26-31.
4. Rohrer D. Interleaving helps students distinguish among similar concepts. *Educ Psychol Rev*. 2012;4:355-367.
5. Kang SHK, Pashler H. Learning painting styles: spacing is advantageous when it promotes discriminative contrast. *App Cognitive Psych*. 2012;26:97-103.

Interleaving Practice Handout:

African Elephant

Image by Matt Artz, retrieved from <https://images.unsplash.com/photo-1503889678302-211ae988b095?ixlib=rb-0.3.5&ixid=eyJhcHBfaWQiOjEyMDd9&s=7ce46670122460d71fac08ed99a6aec6&auto=format&fit=crop&w=1952&q=80> on 10/24/18. Creative Commons License associated: https://creativecommons.org/publicdomain/zero/1.0/

Asian Elephant

Image by Saray Jimenez, retrieved from <https://images.unsplash.com/photo-1513863525216-babdb36af01c?ixlib=rb-0.3.5&ixid=eyJhcHBfaWQiOjEyMDd9&s=b6c59f9152229d2ab3514fb598dad594&auto=format&fit=crop&w=3140&q=80> on 10/24/18. Creative Commons License associated: https://creativecommons.org/publicdomain/zero/1.0/

Interleaving Practice Handout:

Forest Elephant


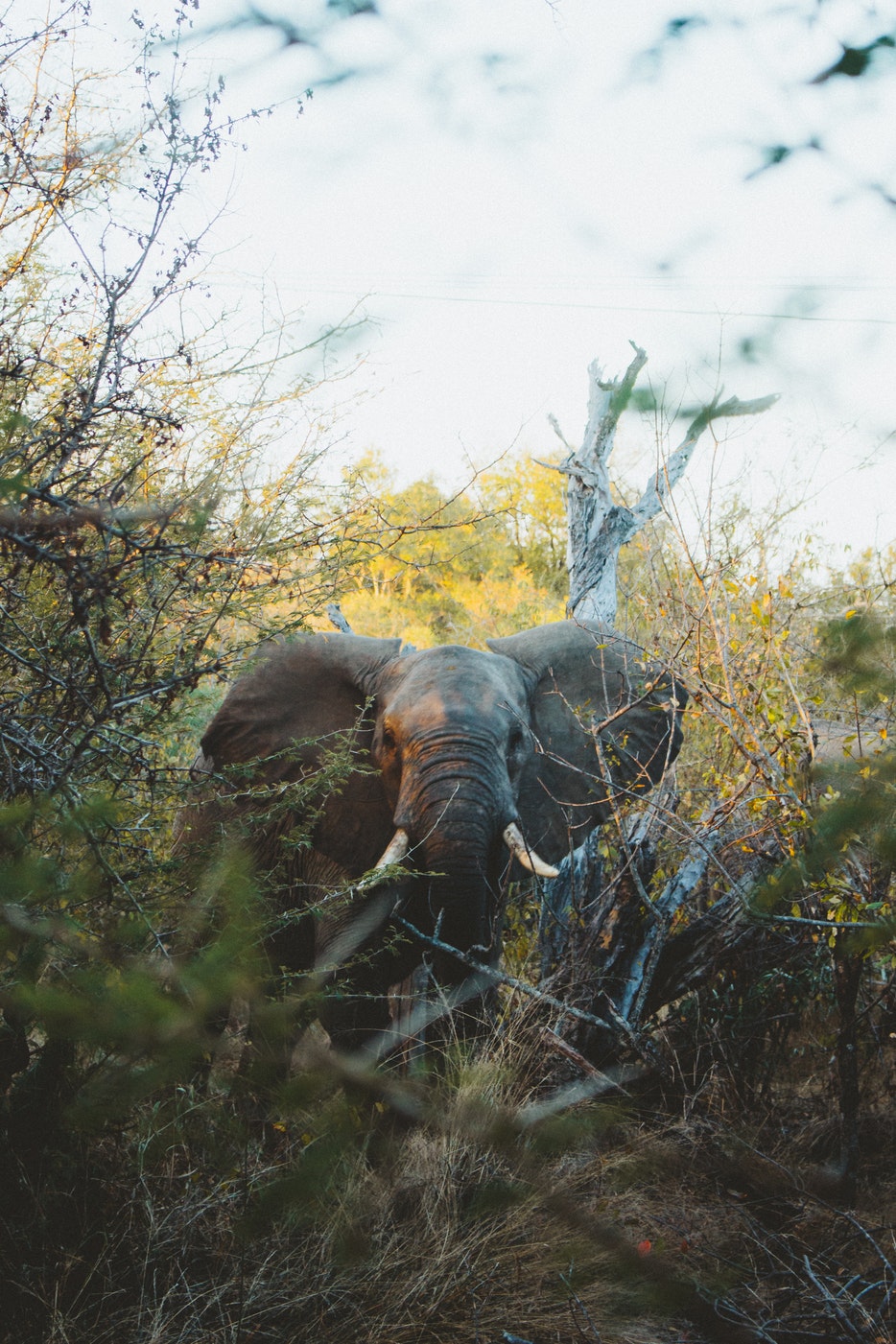


Image by Graham Hunt, retrieved from <https://images.unsplash.com/photo-1516786549992-7fc60748b3a3?ixlib=rb-0.3.5&ixid=eyJhcHBfaWQiOjEyMDd9&s=bfd032206cc2c3825c7284be692c8fc4&auto=format&fit=crop&w=934&q=80> on 10/24/18. Creative Commons License associated: https://creativecommons.org/publicdomain/zero/1.0

Image by Graham Hunt, retrieved from <https://images.unsplash.com/photo-1516786549992-7fc60748b3a3?ixlib=rb-0.3.5&ixid=eyJhcHBfaWQiOjEyMDd9&s=bfd032206cc2c3825c7284be692c8fc4&auto=format&fit=crop&w=934&q=80> on 10/24/18. Image is in the public domain
