# Supplementary material for: Applying Cognitive Learning Strategies to Enhance Learning and Retention in Clinical Teaching Settings
Source: MedEdPORTAL. 2019 Nov 1;15:10850. doi: 10.15766/mep_2374-8265.10850 (PMC6946583; doi:10.15766/mep_2374-8265.10850)
Supplement: Supplementary file 1 — A. Handouts.docx B. Introduction Slides.pptx C. Spaced Retrieval Practice Facilitator Guide.docx D. Interleaving Facilitator Guide and Handout.docx E. Elaboration Facilitator Guide and Handout.docx F. Generation Facilitator Guide and Handout.docx G. Reflection Facilitator Guide and Handout.docx H. Commitment-to-Change Initial Form.docx I. Commitment-to-Change Follow-up Form.docx [file mep-15-10850-s001.zip › E. Elaboration Facilitator Guide and Handout.docx]

**Elaboration Small Group Session:**

User Guide:

- Suggested facilitator wording is noted in quotations

- Instructions are noted in italics

Background on Principle (*1 minute)*:

“The strategy of elaboration connects information to be learned with information that students already know. Putting new information or ideas into pre-existing conceptual frameworks promotes the learning process. In doing so, learners strengthen cognitive connections by extracting key ideas from new material and organizing them into their existing mental frameworks. Students solidify this connection by expressing new knowledge in their own words, creating stronger, longer-lasting memory.”^1,2^

“Why is this important? Researchers have found that the difference between experts’ and novices’ organization of knowledge is the number of connections among the concepts, information, and skills they know.^3^ Experts have dense connections interlacing the many things they know. In contrast, new learners tend to have information, ideas, or skills lodged in their minds in discrete, isolated places. While teachers can help give students suggestions on how to make connections and organize new knowledge into existing mental models, true learning occurs when students make new connections on their own.^4^

Activity (*5 minutes*):

“We’re now going to model how using elaboration can help learners understand new concepts and create lasting memory. Please feel free to write down answers in your handout. *Distribute page 3.*

*“*Who remembers the three methods of heat conduction that you learned in high school chemistry?”

*Pause and let people think and raise their hands. Call on people once they’ve had a time to think until Conduction, Convection, and Radiation have all been named.*

“Great, now who remembers what conduction is?”

*Call on a participant or two to allow answers.*

“Good! For those of you who think you aren’t as familiar, I bet you’re more familiar than you think! We all know how wonderful it feels to hold a warm hot chocolate on a cold winter day. That’s conduction, the first method of heat transfer. Heat transfers through physical touch. Now, who can give me another example of conduction?”

*Call on a participant or two to allow answers.*

“Now, who knows what convection is?”

*Call on a participant or two to allow answers.*

“Great! Similar to conduction, there are very common examples of convection in our everyday lives. For example, everyone is familiar with the phrase “heat rises” or how the basement is the coolest room in your home and the attic always the hottest. That’s because of convection! Warm air rises and carries heat with it before cooling off and falling back down. Now, who can give me another example of convection?”

*Call on a participant or two to allow answers.*

“Lastly, who can explain radiation?”

*Call on a participant or two to allow answers.*

“Exactly! Radiation is heat travel through space. Everyone knows how wonderful it feels to place your hands up to a warm fire on a cold autumn night or how the sun feels on a hot summer day. Now, who can give me another example of radiation?”

*Call on a participant or two to allow answers.*

“Three methods of heat transfer: Conduction, convection, radiation. Three near-universal examples of these experiences in our own lives: Holding a warm glass of hot cocoa, walking into your hot attic, placing your hands up to a warm fire. By connecting an academic concept to things you already know and then having you put the ideas into your own words we are practicing the concept of elaboration and creating more durable memory and mastery.”

Brainstorm activity of how the group can use this skill in their teaching settings (*4 minutes*):

“Now if I’d like everyone to think about how you might use the concept of Elaboration in your own teaching or clinical settings.*”*

*Try to hear as many suggestions as time allows.*

**FOLKMAN (FRONT) | FOLKMAN (BACK) | SEMINAR 1 | BYERS A (RIGHT) | BYERS B (LEFT)**

Reference:

1. Schwartz BL. *Memory: Foundations and Applications*. Thousand Oaks, CA: Sage Publications, Inc; 2011.
2. Brown PC, Roediger HL, McDaniel MA. *Make it Stick: The Science of Successful Learning*. Cambridge, MA: Belknap Press; 2014.
3. Ambrose SA, Bridge MW, DiPietro M, Lovett MC, Foreman MK. *How Learning Works: Seven Researched-Based Principles for Smart Teaching*. San Franciso, CA: John Wiley and Sons; 2010.
4. Lang J. Small changes in teaching: making connections – 3 ways that faculty members can help students link course content to the world around them. *Chron Higher Ed.* <https://www.chronicle.com/article/Small-Changes-in-Teaching-/235230?cid=cp44>. February 8, 2016. Accessed December 18, 2018.

Elaboration Practice Handout:

***Elaboration is the process of giving new information meaning by connecting it with what you already know and expressing it in your own words.***

What are the three methods of heat transfer? What are some examples?

Brainstorming activity: How might you use the concept of elaboration in your own teaching or clinical setting?
