# Supplementary material for: Applying Cognitive Learning Strategies to Enhance Learning and Retention in Clinical Teaching Settings
Source: MedEdPORTAL. 2019 Nov 1;15:10850. doi: 10.15766/mep_2374-8265.10850 (PMC6946583; doi:10.15766/mep_2374-8265.10850)
Supplement: Supplementary file 1 — A. Handouts.docx B. Introduction Slides.pptx C. Spaced Retrieval Practice Facilitator Guide.docx D. Interleaving Facilitator Guide and Handout.docx E. Elaboration Facilitator Guide and Handout.docx F. Generation Facilitator Guide and Handout.docx G. Reflection Facilitator Guide and Handout.docx H. Commitment-to-Change Initial Form.docx I. Commitment-to-Change Follow-up Form.docx [file mep-15-10850-s001.zip › F. Generation Facilitator Guide and Handout.docx]

**Generation Small Group Session:**

User Guide:

- Suggested facilitator wording is noted in quotations

- Instructions are noted in italics

- It is important to note that this exercise requires preparation to choose an uncommon object to present to the participants. However, please feel free to use the enclosed handout, which is a photo of an aquarium air pump.

Background on Principle (*1 minute)*

“Students are much more likely to remember a correct answer if they try to answer a question, construct a solution, or take a guess at a problem on their own before reading the answer or hearing the solution from an instructor. This is known as the generation effect.^1,2^ Research has shown that this effect happens even if students guess the wrong answer; once the actual answer is revealed to them, they will recall the correct answer at a later date.^3^ Why is the generation effect so powerful? By searching their memory or puzzling through a problem, students actively engage in effortful thinking. Spending time and energy generating an answer helps embed information.”^4^

Activity *(6 minutes):*

“Now I am going to have you experience ‘generation’ by having you look at an object and write down what you think the object is and what it is used for. If you know the answer, just write it down; please don't call it out. Remember, it is better to attempt an answer than not to make any attempt.”

*Show a slide of an unfamiliar object or pass the object around (preferable). We used an aquarium air pump (See example on page 3).*

*Point out important characteristics of the object. For example, for the aquarium air pump, you might say,* “Please notice the dome shape, the rubber feet, and the hole on the side of the device.”

*After each person has had a chance to look at the object, ask,* “Does anyone know what this device is and what it's used for? If you know what it is, please still don't say. I would first like to hear from people who have come up with their best guesses.” *Collect answers.*

*Explain what the object is:* “This is an Aquarium Air Pump. It's used to create bubbles to oxygenate the water, which is good for your fish and tank. The dome shape reduces sound and the rubber feet stop it from vibrating and moving.”

“Let's talk about how generation works to help us recall a correct answer at a later time. Researchers believe as you search your mind for an answer to a question or solution to a problem, you encourage deep processing of the question. In doing so you strengthen connections or ‘retrieval routes’ to already existing knowledge in your memory.^5^ For example, you may have thought, ‘I see this device is dome shaped or has rubber feet or a hole in its side.’ Those thoughts create retrieval routes to other devices that you know of with similar features. Once you are given the solution, a light bulb goes on, satisfying your curiosity and establishing a strong cognitive pathway to the answer and connecting the answer to existing knowledge.^4^ You are now much more likely to remember this device if I asked you in a few months than if I just stood here and said, ‘This is an air pump for your aquarium, now let's look at the next aquarium device.’

How do we use Generation in clinical teaching?”

*Provide participants with a handout showing a relevant clinical pathway. As an example, we used the purine metabolism pathway resulting in uric acid and its downstream products. Of note, in our example, the names of the enzymes in the pathway were omitted. See page 4.*

*Explain to the group that tumor lysis syndrome is a disease process that most medical providers encounter at some point in their classroom or clinical experiences. Review that the accumulation of uric acid in the body is one of the main issues in this disease.*

*After each person has a chance to review the pathway, ask*, “If you could design an ideal medication to reduce uric acid in the body, how would it act?” *Collect answers*.

*Summarize the answers as follows*, “There are two ways to decrease uric acid in the body, either by decreasing uric acid production or by increasing uric acid excretion. These are the mechanisms of action of the medications used in tumor lysis syndrome. Allopurinol inhibits xanthine oxidase, the enzyme responsible for uric acid production. Rasburicase potentiates urate oxidase, leading to increased urinary excretion of uric acid.”

*Next explain*: “By generating the mechanisms of these drugs, learners are more likely to retain their effects than if they were simply asked to memorize the drug names and their mechanisms of action.”

Brainstorm activity of how the group can use this skill in their teaching settings (*3 minutes*):

“Now I’d like everyone to think about how you might use the concept of spaced practice in your own teaching setting and have some volunteers share their thoughts with the group. I would also be happy to take any questions about spaced practice at this time.”

*Try to hear at least 2 suggestions before you move on to the next group.*

References:

1. Bertsch S, Pesta B, Wiscott R, McDaniel M. The generation effect: a meta-analytic review.

*Mem Cognition.* 2007;35(2):201-210.

2. Schwartz BL. *Memory: Foundations and Applications*. Thousand Oaks, CA: Sage Publication, Inc; 2011.

3. Yan VX, Garcia MA, Bjork RA. Why does guessing incorrectly enhance, rather than impair retention? *Mem Cognition.* 2014; 42:1373-1383.

4. Brown PC, Roediger HL, McDaniel MA. *Make it Stick: The Science of Successful Learning.* Cambridge, MA: Belknap Press; 2014.

5. Richland LE, Kornell N, Kao LS. The pretesting effect: do unsuccessful retrieval attempts enhance learning? *J Exp Psychol-Appl*. 2009;15(3):243-257.

Author Owned

**FOLKMAN (FRONT) | FOLKMAN (BACK) | SEMINAR 1 | BYERS A (RIGHT) | BYERS B (LEFT)**

Purine Catabolism

Hypoxanthine

Xanthine

Uric Acid

Allantoin

**BAD**

XO

XO

UO

Rasburicase

Allopurinol
