# Supplementary material for: Applying Cognitive Learning Strategies to Enhance Learning and Retention in Clinical Teaching Settings
Source: MedEdPORTAL. 2019 Nov 1;15:10850. doi: 10.15766/mep_2374-8265.10850 (PMC6946583; doi:10.15766/mep_2374-8265.10850)
Supplement: Supplementary file 1 — A. Handouts.docx B. Introduction Slides.pptx C. Spaced Retrieval Practice Facilitator Guide.docx D. Interleaving Facilitator Guide and Handout.docx E. Elaboration Facilitator Guide and Handout.docx F. Generation Facilitator Guide and Handout.docx G. Reflection Facilitator Guide and Handout.docx H. Commitment-to-Change Initial Form.docx I. Commitment-to-Change Follow-up Form.docx [file mep-15-10850-s001.zip › G. Reflection Facilitator Guide and Handout.docx]

**Reflection Small Group Session**

User Guide:

- Suggested facilitator wording is noted in quotations

- Instructions are noted in italics

Background on Strategy (*2 minute)*:

“Reflection refers to reviewing the knowledge, skills and/or attitudes you learned, as a way to consolidate your learning.

Here’s an example of reflection described in the literature and referenced in the book *Make it Stick: The Science of Successful Learning*.^1^ An exercise called ‘write to learn’ is gaining popularity in college classrooms. At the end of class, students are asked to reflect on a concept that they learned in class in a short writing exercise. During this exercise they are asked to: 1) explain the main concept, and 2) describe how it relates to other things they have learned inside or outside the classroom. This exercise was studied with 800 students in a college psychiatry class during which they were asked to ‘write to learn’ on half the content taught over a semester (as compared to receiving summary information for the other half of the content). The students scored a half a letter grade higher on the material they had reflected on.^2^

Using reflection as a way to consolidate learning has also been shown beneficial in learning medicine. For example, a narrative medicine writing exercise included as a part of a geriatric curriculum for internal medicine interns led to increased knowledge retention.”^3^

Activity (*5 minutes*):

“Now I am going to have you use a similar tool and reflect on what you have learned in the session so far today—either from the introductory portion of the workshop or at one of the small group sessions on the other cognitive learning strategies. This method of reflection, developed by Dietz-Uhler and Lanter, is called the ‘Four-Questions Technique.’”^4^

*Hand out worksheet on page 3 and give them 4 minutes to complete on their own time.*

Brainstorm how you could use this skill in your teaching setting (*3 minutes*):

“Now if I’d like everyone to think about how you might use the concept of Reflection in your own teaching setting.”

*Try to hear as many suggestions as time allows.*

**References:**

1. Brown PC, Roediger HL, McDaniel MA. *Make it Stick: The Science of Successful Learning*. Cambridge,MA: Belknap Press; 2014.

2. Gingerich KJ, Bugg JM, Doe SR., Rowland CA, Richards TL, Tompkins SA, McDaniel MA. Active processing via write-to-learn assignments: learning and retention benefits in introductory psychology. *Teach Psychol*. 2014;41: 303–308

3. Maurer MS, Costley AW, Miller PA, McCabe S, Dubin S, Cheng H, et al. The Columbia Cooperative Aging Program: an interdisciplinary and interdepartmental approach to geriatric education for medical interns. *J Am Geriatr Soc*. 2006;54(3):520-6. Erratum in: *J Am Geriatr Soc*. 2006;54(9):1479.

4. Dietz-Uhler B, Lanter JR. Using the four-questions technique to enhance learning. Teach Psychol.. 2009;36(1):38-41.

**Reflection Using the Four-Questions Technique**

1. Identify one important concept, research finding, theory, or idea that you learned while completing this activity. (*analyzing*)
2. Why do you believe that this concept, research finding, theory, or idea is important? (*reflecting*)
3. Apply what you have learned from this activity to some aspect of your life. (*relating*)

4.     What question(s) has the activity raised for you?  What are you still wondering about? (*questioning*).
