# Supplementary material for: Applying Cognitive Learning Strategies to Enhance Learning and Retention in Clinical Teaching Settings
Source: MedEdPORTAL. 2019 Nov 1;15:10850. doi: 10.15766/mep_2374-8265.10850 (PMC6946583; doi:10.15766/mep_2374-8265.10850)
Supplement: Supplementary file 1 — A. Handouts.docx B. Introduction Slides.pptx C. Spaced Retrieval Practice Facilitator Guide.docx D. Interleaving Facilitator Guide and Handout.docx E. Elaboration Facilitator Guide and Handout.docx F. Generation Facilitator Guide and Handout.docx G. Reflection Facilitator Guide and Handout.docx H. Commitment-to-Change Initial Form.docx I. Commitment-to-Change Follow-up Form.docx [file mep-15-10850-s001.zip › H. Commitment-to-Change Initial Form.docx]

1. As a result of participating in this workshop, will you make changes in your teaching (circle one)?

**Yes** (go to question 2) **No** (turn survey in without completing survey)

1. Please describe the change(s) you plan to implement.
2. Please circle your level of commitment in implementing this change.

1 2 3 4 5

Lowest Highest

1. Please list your email address so that we can email you later with a reminder of your commitment to change: _____________________________________________
