# Supplementary material for: Applying Cognitive Learning Strategies to Enhance Learning and Retention in Clinical Teaching Settings
Source: MedEdPORTAL. 2019 Nov 1;15:10850. doi: 10.15766/mep_2374-8265.10850 (PMC6946583; doi:10.15766/mep_2374-8265.10850)
Supplement: Supplementary file 1 — A. Handouts.docx B. Introduction Slides.pptx C. Spaced Retrieval Practice Facilitator Guide.docx D. Interleaving Facilitator Guide and Handout.docx E. Elaboration Facilitator Guide and Handout.docx F. Generation Facilitator Guide and Handout.docx G. Reflection Facilitator Guide and Handout.docx H. Commitment-to-Change Initial Form.docx I. Commitment-to-Change Follow-up Form.docx [file mep-15-10850-s001.zip › I. Commitment-to-Change Follow-up Form.docx]

**User Guide:**

**-** We emailed this evaluation tool as an online survey. We believe it can be adapted to any online survey technology; therefore, for ease of use, we have included the content as a Word document.

-Please find below sample text that can be used to introduce the survey (page 2). We also suggest including the definitions of the cognitive learning strategies at the beginning of the online survey. This text and form can be adapted to the needs of the user.

_____________________________________________________________________________________

It is hard to believe that it has been 6 weeks since our workshop, “Applying Cognitive Learning Strategies to Enhance Learning and Retention in Clinical Teaching Settings.”

We hope you have been practicing many of the cognitive learning strategies since then! At the end of the session, you completed a “Commitment to Change” exercise that is attached to this email. We would love for you to review your responses and then complete this short survey to let us know if you were able to implement any changes as a result of the workshop.

 As a reminder, the 5 cognitive learning strategies that we discussed were:

**Spaced Retrieval Practice** (acronym exercise): Studying information more than once but leaving considerable time between practice sessions.

**Elaboration** (heat transfer exercise): The process of giving new material meaning by expressing it in your own words or connecting it with what you already know.

**Interleaving** (elephant exercise)**:** A method of learning more than one concept at a time so that you can alternate between different problems that call for different solutions.

**Generation** (aquarium pump and tumor lysis exercise)**:** The act of trying to answer a question or solve a problem before being presented any cues, information, or solutions.

**Reflection** (write-to-learn exercise)**:** The act of reviewing what has just been learned as a tool for consolidating knowledge.”

Survey Link: *****************

**Online Survey**

The 5 cognitive learning strategies taught during the workshop were:

**Spaced Retrieval Practice** (acronym exercise): Studying information more than once but leaving considerable time between practice sessions.

**Elaboration** (heat transfer exercise): The process of giving new material meaning by expressing it in your own words or connecting it with what you already know.

**Interleaving** (elephant exercise)**:** A method of learning more than one concept at a time so that you can alternate between different problems that call for different solutions.

**Generation** (aquarium pump and tumor lysis exercise)**:** The act of trying to answer a question or solve a problem before being presented any cues, information, or solutions.

**Reflection** (write to learn exercise)**:** The act of reviewing what has just been learned as a tool for consolidating knowledge.

1. Since you participated in the workshop, have you implemented one or more changes that you listed in the scanned document? (circle one)

**Yes** **No**

1. Since you participated in the workshop, have you implemented one or more changes that you did NOT list in the scanned document? (circle one)

**Yes** **No**

1. *(If yes to either question 1 or 2)* Please briefly describe any changes/teaching strategies that you implemented because of the workshop:
2. *(If no to both question 1 and 2)* Please describe any barriers you encountered to implementing change:
